# Gender differences in LDL- and HDL-cholesterol subfractions in patients after the acute ischemic stroke and their association with oxidative stress markers

**DOI:** 10.3164/jcbn.17-105

**Published:** 2018-03-30

**Authors:** Ingrid Žitňanová, Pavel Šiarnik, Matej Füllöp, Stanislav Oravec, Adela Penesová, Zdenka Ďuračková, Eva Vaská, Peter Turčáni, Branislav Kollár

**Affiliations:** 1 Institute of Medical Chemistry, Biochemistry and Clinical Biochemistry, Faculty of Medicine, Comenius University, Sasinskova 2, 81108 Bratislava, Slovakia; 2 1st Department of Neurology, Faculty of Medicine, Comenius University, Mickiewiczova 13, 81369 Bratislava, Slovakia; 3 1st Department of Internal Medicine, Faculty of Medicine, Comenius University, Mickiewiczova 13, 81369 Bratislava, Slovakia; 4 Institute for Clinical and Translational Research, Biomedical Research Center, Slovak Academy of Sciences, Bratislava, Slovakia; 5 Institute of Physiotherapy, Balneology and Medical Rehabilitation – Piešťany, University of SS. Cyril and Methodius, Trnava, Slovakia; 6 Department of Physical Medicine and Rehabilitation, University Hospital Staré Mesto in Bratislava, Slovakia

**Keywords:** ischemic stroke, small dense-LDL-subfractions, HDL-subfractions, oxidative stress

## Abstract

The aim of our study was to examine gender differences of LDL- and HDL-cholesterol subfractions in patients after the acute ischemic stroke with focus on small LDL and HDL subfractions, and their association with oxidative stress markers. In addition, we have monitored the 7-day effect of cholesterol-lowering drugs administered to patients after the acute ischemic stroke, on these subfractions. Eighty two stroke patients and 81 age matched controls were included in this study. Blood was collected from patients within 24 h after the stroke (group A) and re-examined at the 7-day follow-up (group B). We have found gender differences in LDL- and HDL-subfractions in stroke patients, lipid-lowering drugs administered to acute ischemic stroke patients significantly reduced all measured parameters of lipoprotein profile. In the group A LDL1 subfraction positively correlated with activity of antioxidant enzymes (superoxide dismutase, catalase, glutathione peroxidase) indicating a protective role of this subfraction. On the contrary, small HDL subfractions positively correlated with lipoperoxide levels and negatively with trolox equivalent antioxidant capacity in plasma suggesting a negative role of these subfractions. In this work we have confirmed the hypothesis of atherogenic properties of small HDL subfractions and anti-atherogenic properties of large LDL1-subfractions.

## Introduction

Stroke is a serious medical problem. It is the third most frequent cause of death worldwide. Ischemic stroke is the most common type of stroke which accounts for about 87% of all stroke cases. Several factors can increase the risk of the stroke including non-modifiable and modifiable factors. Non-modifiable factors are gender, race, age, and heredity while modifiable factors are e.g. hypertension, diabetes mellitus, obesity, cardiac problems, alcohol abuse, cigarette smoking, lifestyle factors and dyslipidemia.^(1)^ Hypercholesterolemia represents a modifiable risk factor for the ischemic stroke. However, not all cholesterol increase represents the risk. Research of lipoproteins and their roles in the development of vascular diseases brought differentiated view on atherogenicity of LDL and HDL and their roles in atherogenesis.^(2)^ This is due to the finding that the LDL and HDL classes in terms of physiological functions in the body, and in view of their roles in atherogenesis, are not homogeneous classes and individual subfractions have different biological effects.^(3,4)^ In the LDL class seven LDL subfractions have been identified (LDL1–LDL7) with different biological effects and different atherogenic potential. The most atherogenic subfractions are small dense LDL subfractions 3–7. Their role in the pathogenesis of coronary sclerosis was confirmed.^(4)^ Reasons for their atherogenicity lies in their low recognition by LDL receptors and easier penetration into the subendothelial space as well as formation of cholesterol deposits.^(5)^

The role of large subfractions LDL1 and LDL2 in the development of vascular diseases is not clear yet. It is assumed that in the class of LDL lipoproteins, the subfractions LDL1 and LDL2 are the least atherogenic. In the HDL class which is a protective part of plasma lipoproteins, three major HDL subfractions were identified: large HDL (HDL1–HDL3), intermediate HDL (HDL4–HDL7) and small (HDL8–HDL10) HDL subclasses.^(6)^ Large and intermediate HDL are considered anti-atherogenic subclasses while atherogenic properties of the small HDL subclass have been the subject of much discussion.^(7)^ Increased level of the small HDL subclass was detected in patients with cardiovascular diseases with an atherogenic lipoprotein phenotype B, as well as in patients with lower extremity artery disease.^(6,7)^

In the pathogenesis of ischemic stroke oxidative stress plays an undeniable role. Reactive oxygen species (ROS) can activate several signaling pathways leading to vascular inflammation in cardiovascular diseases: from the initiation of formation of fatty streaks through the progress of lesions to the rupture of ultimate plaques. Reactive oxygen species can also participate in the brain injury after ischemic stroke.^(8)^

The aim of our study was to examine gender differences of LDL- and HDL-cholesterol subfractions in patients after the acute ischemic stroke (AIS) with focus on small, dense, atherogenic LDL and HDL subfractions, and their association with oxidative stress markers. In addition, we have monitored the 7-day effect of cholesterol-lowering drugs administered to patients after the AIS, on these subfractions.

## Material and methods

### Subjects

82 patients (age 68.70 ± 15.90 years) (males/females 46/36) with AIS were included in this study. The control group comprised 81 age matched (64.91 ± 9.01 years) individuals (males/females 36/45) who have never experienced any stroke attacks and showed no signs of acute or chronic diseases. Blood was collected from patients within 24 h after the AIS (group A) and re-examined at the 7-day follow-up (group B). Patients with AIS were taking lipid lowering drugs-statins (atorvastatin 80 mg/day). Samples from all study participants were collected at the 1st Department of Neurology, University Hospital in Bratislava, Slovakia. To determine the subtype of stroke, clinical examination followed by complete paraclinical examination was done. All participants in our study signed an informed consent. This study was approved by the local ethics committee.

### Blood samples

Fasting venous blood was collected from each patient within 24 h after the AIS (group A) and at the 7-day follow-up (group B). From control individuals blood was collected only once after overnight fasting (group Co). Blood samples without anticoagulants were centrifuged for 5 min at 1,200 × *g*, at 4°C and serum was stored in aliquots at −70°C for lipid profile determination.

### Examined parameters

Total cholesterol (TC), LDL-cholesterol, HDL-cholesterol, triacylglycerols (TAG) in serum were determined in the certified laboratory. For the analysis of plasma lipoprotein subfractions, an electrophoresis on polyacrylamide gel-the Lipoprint system, (Quantimetrix corp., Redondo Beach, CA) was used. Oxidative stress markers used for correlation with our results were detected as described by Zitnanova *et al.*^(9)^

### Statistical analyses

The statistical analyses were performed using SPSS ver. 18 (SPSS Inc., Chicago, IL). Significance level was set at *p*<0.05. Since our data were not normally distributed, median was used with interquartile range (IQR), minimal and maximal values. To compare groups Mann-Whitney test was used for particular variables. Spearman correlation coefficients were used to determine relationships among particular parameters.

## Results

Table 1 shows the levels of LDL and HDL subfractions in all experimental groups related to the gender of volunteers involved in our project. In Table 2 there are indicated significant differences between experimental groups.

### Effect of stroke on LDL cholesterol and its subfractions (Table 1 and 2)

Patients (M + F) after AIS, group A (24 h after the AIS) compared to controls had significantly reduced levels of LDL-cholesterol compared to controls due to its significant reduction in the female group, while in males this parameter was not significantly different. They also had no statistically significant changes in small dense LDL subfractions 3–7 compared to controls (M + F). However, when examining these subfraction levels in different genders we have observed their significant elevation only in the male group A. Males in the group A had significantly higher levels of LDL cholesterol compared to females in the same group, while in controls females had higher levels of LDL cholesterol than males. Similarly, subfractions LDL2 and LDL3–7 were significantly higher in males compared to females in A group of patients. Individuals in the group A had significantly higher levels of LDL cholesterol and all its subfractions LDL1–7 compared to experimental group B (7-day follow-up). This increase was observed in both genders. In the group B there was no significant difference in LDL-cholesterol and its subfractions between genders.

### Effect of stroke on HDL-cholesterol and its subfractions

Patients after AIS, group A (24 h after the AIS) had significantly reduced HDL-cholesterol levels compared to controls (M + F). They also had HDL-cholesterol significantly higher compared to the group B. The HDL-cholesterol reduction in the group B was observed in both genders. In addition, they had the large and imHDL subfractions unchanged compared to controls. The same situation was observed in both gender groups. Patients in the group A had significantly reduced small HDL subfractions compared to controls in both genders. There were no significant differences in small HDL subfractions between genders. They also had small HDL subfractions significantly higher in both, the male and also the female groups than in the experimental group B. Moreover, they had levels of HDL, large HDL and imHDL-cholesterol significantly higher compared to the group B. When examining these subfractions in different genders we have observed significantly higher levels of these subfractions only in the female group, while in males large HDL-cholesterol levels were not different between these two experimental groups.

### Effect of stroke on VLDL-cholesterol

Patients after AIS, group A (24 h after the AIS) had significantly reduced VLDL levels compared to controls. This reduction was caused by the significant decline of VLDL levels in the female group. Decrease of VLDL cholesterol in the male group A was not significant when compared to controls. Patients in the group A had no significant change of VLDL-cholesterol levels compared to the group B. They also had significantly increased levels of VLDL-cholesterol in males compared to females, which persisted also in the group B.

### Effect of gender on LDL and HDL subfractions

Males in the group A had total LDL levels similar to those in the male controls but significantly higher than males in the group B. LDL subfractions were similar to those in the controls except LDL3–7 which were higher in males in the group A than in the controls and in the group B. Males in the group A had small HDL subfractions significantly reduced compared to male controls. Moreover, they had significantly lower levels of both, the HDL-cholesterol and large HDL subfractions compared to females. Other HDL subfractions (imHDL and small HDL) were not significantly different between genders.

Females in the group A had total LDL levels significantly lower compared to controls but significantly higher than in the group B. In addition, LDL subfractions were similar to those in the controls. On the other hand, total HDL and small HDL subfractions were significantly reduced compared to female controls.

### Effect of lipid lowering drugs on LDL and HDL subfractions and VLDL (group B)

Patients in the experimental group B (M + F) had significantly lower levels of all LDL and HDL subfractions compared to the group A except of the small HDL subfractions which were nonsignificantly reduced. Lipid lowering drugs significantly reduced the small dense LDL3–7 subfractions in males and small HDL subfractions in both genders. However, 7-day administration of lipid lowering drugs had no effect on VLDL levels.

### Correlations of lipid parameters with oxidative stress markers

Significant correlations between lipid parameters and markers of oxidative status are reported in Table 3. Nonsignificant correlations are not shown.

## Discussion

Several studies have clearly shown that plasma LDL-cholesterol, along with HDL-cholesterol and TAG concentrations together with other genetic factors are closely linked to vascular diseases.^(10,11)^ In their development also oxidative stress plays an important role. In this project we have examined gender differences of LDL- and HDL-subfractions in patients after the AIS and their association with oxidative stress markers. In addition, we have monitored the 7-day effect of cholesterol lowering drugs administered to patients after the AIS on these subfractions.

### Effect of stroke on LDL and HDL subfractions

We have found that patients after the AIS in the group A (24 h after the AIS) had significantly reduced levels of total LDL-cholesterol compared to controls. This reduction was more pronounced in the female group. In addition, except the HDL-cholesterol levels which were significantly higher in the control group than in the group A, controls had more atherogenic lipoprotein profile compared to the age-matched patients in the group A. Specifically, controls had significantly greater total LDL, total HDL, small HDL-subfraction as well as VLDL-cholesterol levels. It indicates a lipid-lowering effect of AIS. Other studies report similar effects of AIS on lipoprotein levels,^(12)^ however the mechanism of this action is unknown yet. It has been suggested that ischemic attack might lead to the overproduction of catecholamine secretion^(13)^ which might result in reduced levels of lipoprotein particles. Similar effects have been observed in patients after myocardial infarction.^(14)^

### Effect of gender on LDL and HDL subfractions

 Plasma lipid levels are generally thought to be regulated by sex hormones leading to sexual dimorphism in the lipid profile. However, the problem seems complicated in older individuals. In patients after AIS group A there were significant differences between genders in LDL lipoprotein subfractions. Males had significantly increased levels of LDL-cholesterol as well as LDL2- and small, dense atherogenic LDL3–7 subfractions compared to females. On the contrary, HDL-cholesterol and large HDL subfractions were significantly elevated in the female group A compared to the male group A. The observed gender differences might not be related to sex hormones because majority of our patients were after the menopause or andropause. Our results are in accord with some earlier studies. Menopause induced surgically without hormone replacement therapy showed no effect on lipoprotein profile compared to hysterectomy with conservation of the ovaries.^(15,16)^ Sexual dimorphism in metabolism of lipoproteins might be a consequence of a complex network of hormone actions combined with sex-specific modulators of lipid metabolism. One of the possible candidate could be insuline with its different action between males and females. This pancreatic hormone is an important regulator of lipid metabolism.^(17,18)^ Results from several studies indicate that dyslipidemia associated traditionally with menopause can be rather caused by chronological aging than by the dysfunction of ovaries.^(19–21)^ There still need to be elucidated underlying physiological modulators of plasma lipid metabolism responsible for the differences between older men and women.

### Effect of lipid lowering drugs on LDL and HDL subfractions

In healthy individuals statins have been shown to mildly increase or not alter HDL-cholesterol and moderately affect LDL subclass distribution.^(22)^ We have focused our attention on small dense atherogenic LDL- and HDL subfractions in patients after the AIS. The 7-day statin administration to our patients surviving the AIS resulted in the significant reduction of not only small dense LDL3–7-subfractions but of all lipid parameters (small HDL and VLDL decrease was non-significant). Moreover, statins abolished significant differences in lipid subfractions between genders. While in AIS patients (group A) significantly different levels of most LDL subfractions (except LDL1) and some HDL subfractions were detected in different genders, 7 days after the AIS with lipid lowering therapy, these differences were abolished in all LDL and HDL subfractions (except the total HDL). Statins have been assumed to have beneficial diverse effects on the organism. These effects are based also on other mechanisms than on the inhibition of HMG-CoA reductase (3-hydroxy-3-methyl-glutaryl-CoA reductase) in the liver. The other beneficial effects include antioxidant effects, infammatory cascade inhibition, plaque stabilization, modulating platelet coagulation and upregulation of NO-synthase with consequent elevation of cerebral blood flow.^(23)^

### Association of lipid profile with markers of oxidative stress

In the group A (males and females) a positive correlation between small HDL-cholesterol subfractions and lipoperoxide levels and a negative correlation between small HDL-cholesterol subfractions and TEAC indicate an atherogenic character of small HDL-cholesterol subfractions. On the other hand, LDL1-cholesterol subfractions always positively correlated with activity of antioxidant enzymes suggesting anti-atherogenic character of LDL1-cholesterol subfraction. These results suggest to include small HDL-cholesterol subfractions to risk factors for atherosclerosis and large LDL1-cholesterol subfraction to protective plasma particles.

Identification of lipoprotein subfraction profile will enable us to distinguish normocholesterolemic individuals with increased risk of premature atherothrombosis. On the other hand, it can be assumed that the increased concentration of LDL1 in plasma of patients with hyperbetalipoproteinemia (elevated levels of LDL-cholesterol in blood) does not always represent an increased risk for the development of coronary diseases.^(6)^ Confirmation of this assumption could have substantial practical applications in the treatment of vascular diseases and lipid metabolism disorders.

Potential limitations of the present study include the absence of the control group related to the experimental group B as well as an uneven representation of female/male ratio in the patient group (males/females 46/36) as well as in controls (males/females 36/45).

The main findings of our experimental study focusing on stroke patients are that there are gender differences in LDL- and HDL-subfractions in spite of the fact that majority of our patients were after menopause or andropause. In addition, we have found significant associations between several vascular disease risk factors and markers of oxidative status and confirmed the hypothesis of atherogenic properties of small HDL subfractions and anti-atherogenic properties of large LDL1-subfractions.

Taking into account our results, new approach to prevention and treatment of vascular diseases should be accepted in clinical practice. Effective assessment of the overall vascular risk should include also the complex analysis of major lipoprotein classes as well as development of sensitive and specific biomarkers to assess the oxidative stress phenotypes. These are new avenues for future research as we move toward the broader use of pharmacological and regenerative therapies in the prevention and treatment of vascular diseases.

## Figures and Tables

**Table 1:** Phenotypic differences in LDL and HDL subfractions and VLDL lipoproteins among males and females in three experimental groups

Parameter (mg/dl)	Controls				AIS A				AIS B		
	M + F	M	F		M + F	M	F		M + F	M	F
LDL	115	109	116		101.5	102	96		82	77	85.5
	(90.5–135)	(83–124)	(95–138)		(85–129)	(84–135)	(87–128)		(71–96)	(71–99)	(65–93.5)
LDL1	40.5	35	46		44	41	45		33	31	34
	(32–53)	(29–42)	(34–56)		(33–51)	(29–49)	(35–51)		(27–37)	(25–37)	(25.5–35.5)
LDL2	19	20	18.5		20	23	15		10	11	8
	(9.5–29.5)	(10–30)	(8–29)		(13–26)	(18–30)	(10–24)		(5–16)	(7–19)	(4–11.5)
LDL3–7	0	0	0		0	2	0		0	0	0
	(0–2)	(0–2)	(0–2)		(0–3)	(0–5)	(0–0)		(0–3)	(0–0)	(0–0)
HDL	43	38	47		40	39	46		33	33	33
	(37–50)	(33–43)	(38–53)		(34–49)	(32–45)	(35–52)		(30–38)	(27–34)	(31.5–39)
large HDL	12.5	10	15		15.5	12	18		11	10	12.5
	(9–18)	(7–13)	(11–20)		(10–19)	(8–16)	(15–23)		(8–16)	(8–12)	(9–18.5)
imHDL	23	21	24		21.5	20	22		19	18	18.5
	(20–26)	(19–24)	(21–28)		(19–26)	(18–25)	(20–27)		(16–21)	(14–24)	(16.5–20)
small HDL	6	6	6		4	4	4		3	3	2.5
	(5–9)	(5–9)	(5–9)		(3–6)	(3–6)	(2–5)		(2–4)	(2–4)	(2–4)
VLDL	32	31	33.5		28	30	20		23	28	19
	(27–41)	(7–41)	(27–42)		(19–36)	(20–38)	(17–31)		(19–32)	(23–32)	(17.5–19)

Values are presented as a median and interquartile range. M, males; F, females; AIS A, group of patients within 24 h after the AIS; AIS B, group of patients 7 days after the AIS; LDL, low-density lipoproteins; HDL, high-density lipoproteins; imHDL, intermediate HDL; VLDL, very low-density lipoproteins.

**Table 2:** Significations (P) in lipid parameters between experimental groups

Signification *p*	M + F			M			F			M vs F	
Parameter (mg/dl)	A/Co	A/B		A/Co	A/B		A/Co	A/B		A(M)/A(F)	B(M)/B(F)
LDL	**0.0268**	**0.0075**		0.3902	**<0.0001**		**0.0002**	**0.0002**		**0.0136**	0.5241
LDL1	0.9777	**<0.0001**		0.5058	**<0.0001**		0.886	**0.0017**		0.2683	0.7298
LDL2	0.4175	**<0.0001**		0.1885	**<0.0001**		0.5075	0.0532		**0.0223**	0.3184
LDL3–7	0.2432	**0.0094**		**0.0217**	**0.0059**		0.1934	0.6557		**0.0014**	0.5852
HDL	**0.006**	**<0.0001**		0.1	**0.0009**		**0.0375**	**0.0225**		**0.0119**	**0.036**
large HDL	0.2069	**0.0043**		0.1592	0.1461		0.1214	**0.0142**		**0.0003**	0.0874
imHDL	0.1158	**0.0033**		0.8154	**0.001**		0.1522	**<0.0001**		0.3139	0.4799
small HDL	**<0.0001**	0.0671		**0.0035**	**0.0205**		**<0.0001**	**0.015**		0.1535	0.6168
VLDL	**0.0171**	0.4296		0.6485	0.1862		**0.0014**	0.7888		**0.0182**	0.0567

M, males; F, females; A, group of patients within 24 h after the AIS; B, group of patients 7 days after the AIS; Co, control individuals; A(M), only male patients in the group A; A(F), only female patients in the group A; LDL, low-density lipoproteins; HDL, high-density lipoproteins; imHDL, intermediate HDL; VLDL, very low-density lipoproteins. Statistically significant differences are indicated in bold.

**Table 3:** Statistical significance (P) of correlations between measured parameters in three experimental groups (controls, group A, group B) using Spearman correlation test

Group A					Controls			
Parameter	Parameter	*p*	*r*		Parameter	Parameter	*p*	*r*
		
Males and females					Males and females			
LDL	catalase	0.0041	+		LDL1	age	0.0081	+
LDL1	SOD	0.0155	+		LDL2	lipoperoxides	0.0255	+
	catalase	0.0393	+		large HDL	SOD	0.0145	+
	GPx	0.002	+		Males			
small HDL	lipoperoxides	0.0071	+		LDL1	TEAC	0.0055	+
	TEAC	0.0006	–		LDL2	lipoperoxides	0.0029	+
Males					LDL3–7	age	0.0203	–
LDL	catalase	0.0016	+		HDL	SOD	0.0055	+
LDL1	catalase	0.0304	+		large HDL	SOD	0.0026	+
	GPx	0.0021	+		imHDL	SOD	0.0273	+
LDL2	catalase	0.0319	+		Females			
large HDL	catalase	0.0117	+		LDL1	SOD	0.0395	+
imHDL	SOD	0.0345	+		LDL3–7	age	0.0444	+
small HDL	TEAC	0.0451	–		large HDL	SOD	0.0483	+
Females								
LDL	lipoperoxides	0.0366	+					
LDL1	SOD	0.0471	+		Group B			
HDL	GPx	0.0336	+		Males			
small HDL	lipoperoxides	0.0204	+		LDL1	catalase	0.0021	+
	TEAC	0.0372	–		large HDL	GPx	0.0193	+

Group A: 24 h after AIS, group B: 7-days after AIS. SOD, superoxide dismutase; GPx, glutathione peroxidase; TEAC, trolox equivalent antioxidant capacity; LDL, low-density lipoproteins; HDL, high-density lipoproteins; imHDL, intermediate HDL. Statistically significant: *p*<0.05.
